# Social Deprivation Index Is Associated with 90-Day Emergency Department Revisits, but Not Admission, for Acute Heart Failure

**DOI:** 10.5811/westjem.61392

**Published:** 2026-07-16

**Authors:** Robert R. Ehrman, Nicholas E. Harrison, Brian D. Haber, Steven J Korzeniewski, Mark J. Favot, Lyudmila Khait, Phillip D. Levy

**Affiliations:** * Wayne State University School of Medicine, Integrative Biosciences Center, Department of Emergency Medicine, Detroit, Michigan; † Indiana University School of Medicine, Department of Emergency Medicine, Indianapolis, Indiana

## Abstract

**Background:**

Prevalence of heart failure is increasing, with an associated rise in emergency department (ED)-related care. Social determinants of health (SDoH) are associated with adverse health outcomes, but the extent to which they influence ED use for heart failure care is poorly understood. We sought to describe the relationship between community-level social vulnerability and ED revisits and hospital admissions for heart failure within a single health system.

**Methods:**

We conducted a retrospective review of Social Deprivation Index (SDI) scores (higher score = more deprivation) by ZIP code paired with administrative clinical data. Zero-hurdle regression was used to model the relationship between SDI and ED revisits within 90 days of a prior ED visit for heart failure (logistic model for > 0 revisits; Poisson model for count of revisits). We used a mixed-effects logistic model—accounting for repeat visits—to test the association of SDI with hospital admission at any given ED visit.

**Results:**

From January 2022–December 2023, there were 3,569 ED visits from 2,406 patients. Each standard deviation increase in SDI (30.3) was associated with increased odds of at least one 90-day revisit (OR, 1.53; 95% CI, 1.08–2.16). Higher SDI was also associated with more 90-day revisits, with varying magnitude by hospital. After adjusting for characteristics of prior ED visit, SDI was not associated with hospital admission.

**Conclusion:**

Our results suggest that area-level social vulnerability influences the decision to seek heart failure-related care in the ED. Patients from more deprived areas may not have more severe clinical presentations, however, as evidenced by lack of association of SDI with hospital admission.

## INTRODUCTION

The number of heart failure-related emergency department (ED) visits is increasing. Data from the early 2000s found they accounted for approximately 1% of all ED visits,^1^ while a more recent study reported an increase to nearly 4%.^2^ Most (70–80%) visits for acute decompensation of heart failure (acute heart failure) result in admission, and 30% of patients are re-admitted within 90 days.^3^ Many of those discharged from the ED have return visits within 90 days.

Heart failure-related ED revisits are driven by multiple factors, including symptoms (eg, dyspnea, peripheral edema) and tangible needs (eg, medication refills). Social determinants of health (SDoH)—the level of education, socioeconomic status, access to reliable transportation, etc—are also likely drivers of real and perceived need to return to the ED. Social determinants of health are known to increase the risk for development of chronic diseases and decrease the likelihood of high-quality disease management, leading to enhanced risk for ED visits and hospital (re)admissions.^4^,^5^

The effects of SDoH, operating at the area-level (neighborhoods, ZIP codes, etc), on overall health outcomes are well-described,^6^,^7^ but the association with heart failure-related ED use is understudied and incompletely understood.^8^,^9^ Therefore, our goal in this study was to assess the association with area-level SDoH and heart failure-related ED revisits within a single health system in Detroit.

## METHODS

### Study Design and Participants

In this retrospective cohort study we used billing data for ED visits that occurred between January 2022–December 2023 at an integrated health system in Detroit, affiliated with Wayne State University (WSU). Eligible patients were those with a primary or secondary ED diagnosis of heart failure who presented to any of the four teaching hospitals within the health system. The study was approved by the WSU Institutional Review Board. Manuscript preparation was in accordance with the Strengthening the Reporting of Observational Studies in Epidemiology (STROBE) guidelines.^10^

### Clinical Setting and Data

Combined annual ED volume at the four hospitals is approximately 150,000 patients. Included are Detroit Receiving Hospital (DRH), an urban academic Level 1 trauma center; Harper University Hospital (HUH), an urban academic tertiary-care center that does not receive 9-1-1 traffic; Sinai-Grace Hospital (SGH), an urban community teaching hospital; and Huron Valley Sinai Hospital (HVSH), a suburban academic-affiliated hospital.

Emergency department-level data available for each visit included the following: date of service; birthdate; biological sex; ED disposition (admit, discharge, left against medical advice (AMA), and death in the ED), home ZIP code; and *International Classification of Diseases, 10^th^ Revision* (I*CD-10*) codes assigned for billing purposes. Admission location (intensive care unit floor) and hospital disposition were not available, nor were patients’ full street addresses. Unique medical record numbers were available for each patient, enabling us to track repeat visits.

### Case Definition

Heart failure was defined by *ICD-10* code I50.x. We excluded patients with co-occurring *ICD-10* codes that may have confounded reason for presentation (eg, ST-elevation myocardial infarction, pneumonia). The full list of exclusions is provided in the Supplement (Table S1, n = 156 patients). We performed chart reviews for a random sample of 25 included, and 25 excluded, patients to confirm the presence or absence of acute heart failure (no erroneous inclusions or exclusions were found during manual adjudication) and to optimize rigor and reproducibility. We excluded 21 patients who died in the ED (18 on index visit and three at repeat visits) because death was outside the scope/focus of this investigation, and the low number of deaths precluded detailed analysis.

**Population Health Research Capsule**

What do we already know about this issue?

*Social vulnerability is associated with adverse health outcomes, but the relationship with emergency department (ED) use for acute heart failure is not well characterized*.

What was the research question?

*Our goal was to determine whether the Social Deprivation Index (SDI) is associated with 90-day ED revisits, or admissions, for heart failure*.

What was the major finding of the study?

*Each standard deviation increase in SDI (30.3) was associated with increased odds of a 90-day revisit (OR, 1.5; 95% CI, 1.08–2.16)*.

How does this improve population health?

*Identifying factors associated with ED visits for heart failure is a critical first step in designing interventions to optimize care and resource allocation*.

### Primary and Secondary Exposures

We extracted area-level data from WSU’s PHOENIX Virtual Data Warehouse.^11^ We chose the Social Deprivation Index (SDI) as our primary exposure of interest because our data included ZIP codes but not full street addresses or ZIP+4. The SDI is a seven-item composite score that includes percentages of the following: living in poverty; attaining < 12 years of education; living in single-parent households; in rented housing units; in overcrowded housing units; in households without a car; and having non-employed adults < 65 years of age. Scores range from 1 (least) to 100 (most deprivation).^12^

Secondary area-level exposures included data at the ZIP code level on the following metrics: extreme income disparities (Index of Concentration at the Extremes, ranging from −1 [least wealth] to 1 [most wealth],^13^ as per the U.S. Census Bureau); lack of health insurance (expressed as percentage of adults 18–64 years of age, as per the U.S. Centers for Disease Control and Prevention: ED use (as ED visits per adult population, based on ED surveillance data); and hypertension prevalence (from local ED surveillance data, using *ICD-10* codes for hypertension; and median systolic blood pressure (from ED surveillance data). The latter two were chosen given the close link between hypertension and development of heart failure and progression of disease, as well as the association between area-level hypertension prevalence and adverse cardiovascular outcomes.^14^,^15^

### Primary and Secondary Outcomes

The primary outcome measure was occurrence of at least one ED-revisit for acute health failure within 90 days of a prior heart failure-related ED visit (Outcome 1). We hypothesized that increased social vulnerability, as measured by SDI, would be associated with greater likelihood of 90-day revisits. This 90-day endpoint was chosen to allow sufficient time for SDoH to manifest themselves (eg, inability to attend a follow-up visit at which medication refills could be provided), as opposed to shorter time frames that may more prominently reflect undertreatment and/or treatment failure at the initial encounter. While the exact time frame over which SDoH have maximal impact on outcomes for acute heart failure is not known, the chosen timepoint includes the early posthospital discharge vulnerable phase,^16^,^17^ when adverse outcomes are greatest for patients with acute health failure. Secondary outcome measures included the per-patient count of 90-day heart-failure revisits (Outcome 2), and overall likelihood of heart failure-related hospital admission for any given heart failure-related ED visit (Outcome 3).

For the purposes of 90-day outcomes, a visit was considered an “index” visit if there were no prior ED visits or admissions within the preceding 90 days. We excluded visits within the first 90 days of the dataset because it was not possible to determine whether these represented index visits or re-visits. Patients with index visits that occurred in the prior 90 days of the dataset were also excluded from 90-day outcome assessment.

Given that the sample size was determined by the amount of available data, no power calculation was performed.

### Statistical Analysis

Descriptive data are reported as medians (with interquartile range [IQR]), or proportions, as appropriate. To account for the excess zeros in the count of 90-day revisits, outcomes 1 and 2 above were modeled using two-step zero-hurdle regression. The primary outcome (0 versus ≥ 1 90-day revisit) was modeled with a logistic model for the zero-hurdle component (ie, likelihood of ≥ 1 versus 0 revisits, Outcome 1) and a positive Poisson model (representing ≥ 1 revisit) was used for the count portion (Outcome 2). Covariates included in all models were SDI, age, biologic sex, and hospital. The additional SDoH variables listed above were also considered for inclusion. We also considered two- and three-way interactions, as well as non-linear transformation of continuous covariates (restricted cubic spline with 5 knots, at quantiles 0.05, 0.275, 0.5, 0.725. and 0.95 [18]). Numerical variables were mean-centered and scaled. Variable selection was performed using Akaike Information Criteria and likelihood ratio testing for nested models. The same covariates were considered for both the zero and count components, with the number of index ED visits included as an offset in the count model. Assessment of over-dispersion in the count data was performed to ensure appropriate use of the Poisson distribution. After final model selection, the following diagnostics were performed on each sub-model: visual inspection of QQ plot; Studentized residual plot; calibration plot (for logistic model); and plots of DFFITS/DFBETAS. We assessed multicollinearity using variance inflation factor.

We used mixed-effects logistic regression to model the likelihood of hospital admission for any given A ED visit related to acute heart failure (Outcome 3). Fixed effects for this model included the following: SDI; age; biologic sex; hospital; days elapsed since prior ED visit (set to 0 for index visits); and disposition at prior ED visit (index, admit, discharge, or AMA). Other area-level factors, as detailed above, were also considered for inclusion. To account for repeat visits we added a random intercept for each patient. Numerical variables were mean-centered and scaled. Contrasts between levels of categorical variables were calculated as estimated marginal means (ie, at mean values for continuous variables and averaged across levels of categorical variables).

We performed sensitivity analyses, excluding patients at HVSH (suburban vs urban location of other hospitals) to test whether this altered the effect of SDI on outcomes. Statistical analysis was performed using RStudio Integrated Development Environment for R v2024.4.2.764 (Posit Software, PBC, Boston, MA). Models were fit with the “glmmTMB” package, and marginal means were calculated using “emmeans” and “mariginaleffects” packages. All models were estimated with maximum likelihood and cluster-robust (by hospital) standard errors. Statistical significance was set at 0.05.

## RESULTS

### Participant Characteristics

**Figure 1:**
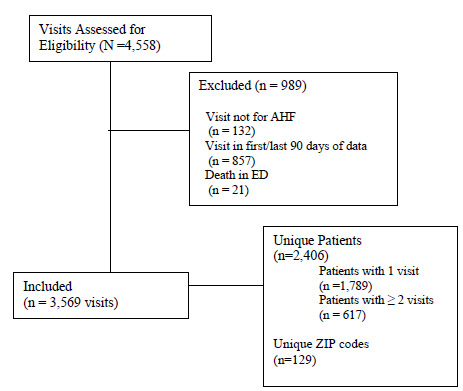
Patient flow diagram in a study describing the relationship between community-level social vulnerability and emergency department revisits and hospital admissions for heart failure. *AHF*, acute heart failure; *ED*, emergency department.

After exclusions (Figure 1), there were 2,406 unique patients from 129 ZIP codes, included for analysis. Approximately half of the participants were female, the median age was 66 (IQR 57–75) years, and the median ZIP-code-level SDI was 94 (IQR 79–96). Additional characteristics are listed in Table 1.

### Visit Characteristics

During the study period, there were 3,569 total ED visits from 2,406 unique patients; 1,789 patients had a single visit and 617 patients had > 1 visit. The median number of ED visits in the full cohort was 1 (IQR 1–2); for patients with > 1 visit, the median ED visit count was 2 (IQR 2–3). For those with > 1 visit, the median time between ED visits was 53.5 (IQR 22–130) days. In patients with single visits, median SDI was 94 (IQR 79–95); for patients with ≥ 1 revisit, median SDI was 95 (IQR 90–96). Median area-level SDI for each hospital was 95 (IQR 95–97) for DRH, 95 (IQR 91–97) for HUH, 91(IQR 90–95) for SGH, and 13.5 (IQR 10–24) for HVSH.

Overall, 72% of ED visits (2,577/3,569) resulted in admission, and 70% of patients (1,683/2,406) had at least one admission. Hospital admission occurred in 72% of index visits (2,042/2,822). Comparison of disposition on the index, versus non-index, visits is shown in Table 2.

### Missing Data

Social Deprivation Index and health insurance data were missing for the same single ZIP code, which resulted in exclusion of one visit from primary and secondary outcome analyses. Median systolic blood pressure was missing for 17 ZIP codes, which resulted in exclusion of 18 and 21 visits from the primary and secondary outcome analyses, respectively. Complete data were available on all other variables.

### Primary and Secondary Outcomes

**Figure 2:**
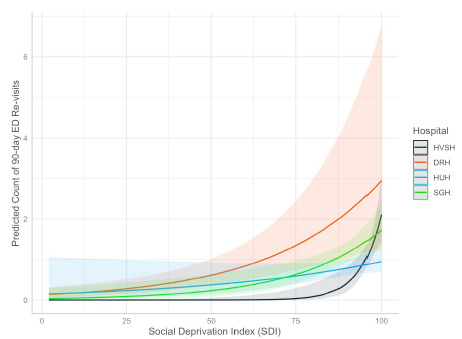
Plot of hospital-Social Deprivation Index interaction for count model in a study describing the relationship between community-level social vulnerability and emergency department revisits and hospital admissions for heart failure. *ED*, emergency department.

Of the 2,406 total patients, 409 (17%) had ≥ 1 re-visit for acute heart failure within 90 days of a prior heart failure visit. For the zero-hurdle component (Outcome 1), each 1 SD increase in SDI (30.3) was associated with increased likelihood of at least one 90-day revisit (OR 1.53; 95% CI, 1.08–2.16). For the count model (Outcome 2)—but not the zero-hurdle model—there was a significant SDI*hospital interaction (*P* = .02). As shown in Figure 2, increasing SDI was associated with greater number of 90-day revisits, but the magnitude varied by hospital. Predicted counts of 90-day revisits at varied levels of SDI are provided in the Supplement (Table S2).

In addition to SDI (and the SDI*hospital interaction in the count component), the final models included age, biological sex, hospital, median systolic blood pressure, and ZIP code-level percentage without health insurance; parameter estimates are provided in Table 3. For the zero-hurdle component, significant differences for biologic sex and hospital were found, but there were no significant associations with age, health insurance status, or median systolic blood pressure. Income disparity was not statistically associated with revisits and worsened model fit; therefore, we excluded it. In the count model, median systolic blood pressure was associated with increased visit count (RR 1.47; 95% CI, 1.17–1.84) per SD (2.07) increase. Other significant associations were age and lack of health insurance (inverse relationships) and biologic sex (more revisits for males).

### Mixed-Effects Model (Outcome 3)

Fixed-effects variables included in the final model were age, biological sex, hospital, days elapsed since prior visit (set to 0 for index visit), and disposition at prior visit (reference category set to Index visit). Best model fit was achieved with inclusion of SDI median systolic blood pressure, and percentage without health insurance, although none achieved statistical significance (Table 4). Increasing age was associated with greater likelihood of admission (OR 1.25; 95% CI, 1.14–1.76 per SD (14.1 years) increase, as was increasing time between visits (OR 1.45; 95% CI, 1.29–1.62) per SD (76.3 days) increase (alternatively, OR 1.13 per 25.4 days). Income disparity was excluded from the final model for the same reasons as with the primary outcome.

The SD of the random intercept was 0.59, which translates to a patient-level effect on the OR of admission at any given visit of +/−1.80 and indicates substantial patient-level variability when compared to fixed-effects estimates (ie, the average effects in the full sample population).

Associations between disposition at prior visit and hospital on the odds of admission, as well as all as all sensitivity analyses, are provided in the Supplement (Figures S1, S2 and Tables S3 and S4).

## DISCUSSION

In this single-center, retrospective study we found that higher SDI score was associated with increased risk and frequency of 90-day ED revisits for heart failure but not with hospital admission. These results suggest that while neighborhood-level social vulnerability is associated with the decision to seek heart failure-related ED care, that vulnerability does not necessarily result in more severe clinical presentations that require hospital admission. Results of the sensitivity analysis, however, suggest that the effect may vary across the spectrum of vulnerability (ie, in areas with high versus low SDI). Additional studies, encompassing the full range of SDI scores, are needed to elucidate the mechanisms through which social vulnerability influences the decision to seek ED care. Such data are critical for the development of interventions to safely reduce ED use among patients with heart failure.

Identification of acute heart failure patients who are safe for discharge from the ED has been studied extensively.^19^ Despite the existence of several reliable risk stratification tools, they remain poorly used.^20^ Equally important, but studied less, are strategies that safely keep patients from visiting the ED. Asthana et al found that a brief, heart failure-focused educational intervention in the ED reduced revisits at 30- and 90-days.^21^ The intervention focused on basics of outpatient heart failure management, as well as suggestions of when to seek outpatient versus ED care. While doubtless critical to long-term disease stability, this approach presupposes that patients have access to a litany of resources (medications, healthy foods, outpatient care, etc), which may not always be the case.

Our results are novel, in that we identified modifiable, community-level risks of increased ED use related to heart failure, but also complementary to those of Asthana et al, in that understanding the geospatial distribution of social vulnerability may allow targeted deployment of interventions based on area-level needs. For example, mobile health units could target highly vulnerable areas to ensure patients receive post-discharge follow-up visits and medication refills.

Another unique aspect of our study is that we explored the association of social vulnerability on ED revisits and hospital admissions (irrespective of whether a visit was an index or a revisit, while accounting for repeat visits by individual patients and disposition at the prior visit). Social determinants of health have been linked to adverse health outcomes^6^,^7^; therefore, a plausible assumption is that the most vulnerable patients would be frequent ED users and likely to be admitted at each visit. However, this was not the case, as increased SDI was associated with ED use but not admission. These results show that careful and nuanced interpretation of such data is warranted so that interventions can be appropriately developed and implemented where need is greatest. The (estimated) patient-level variability on the odds ratio of admission (+/− 1.80) likewise highlights the importance of collection of robust, patient-level measures of vulnerability owing to heterogeneity of treatment effect (whereby composite scores may not be equally associative for all patients).

We found that among patients with at least one 90-day revisit, greater mean systolic blood pressure was associated with more ED revisits (RR 1.47; 95% CI, 1.17–1.84). Given the relationship between lack of blood pressure control and disease progression, patients from ZIP codes with higher mean systolic blood pressure may represent a “sicker” population and, thus, potentially more likely to have heart failure-related ED visits. Moreover, because systolic blood pressure is such an important risk factor for heart failure, it also represents a potential quality-of-care indicator with worse population-level control, reflecting challenges such as poor access to care or inability (or unwillingness) to adhere to a prescribed diet/medication regimen. While these explanations reflect the reasoning for inclusion of this covariate in our models, further study is needed to delineate the relationship between mean systolic blood pressure and heart failure outcomes.

The finding that the association between SDI and number of 90-day revisits varied by hospital, and with larger magnitude at higher SDI scores, may be due to patient- and/or hospital/physician-level factors. Elucidating causal effects will require populations with greater variation of SDI scores (particularly in the mid-range), consideration of patient clustering within-physicians and collection of patient-level data.

Increased age and proportion with health insurance were both negatively associated with fewer ED revisits (RR 0.68; 95% CI, 0.60–0.76, and 0.64; 95% CI, 0.59–0.84, respectively). Younger patients may be those with a newer heart failure diagnosis and, owing to lesser duration of disease, they may not yet have acquired health insurance or established relationships with outpatient clinicians, thereby resulting in more ED use. Existing studies suggest that, overall, those with health insurance tend to use the ED more often than the uninsured,^22^ although robust heart failure-specific data are notably lacking. In contrast, other SDoH, including non-Hispanic Black race, low educational attainment, and low household income, have been associated with more ED use for heart failure.^23^,^24^ This highlights the need for more detailed investigation in this area as both individual- and community-level needs and behaviors likely influence the decision to seek ED care.

## LIMITATIONS

There are several limitations to our results that warrant discussion. Our data may not generalize to other locations and populations, the observational nature of our study precludes determination of causation versus association, and area-level data are subject to the ecologic fallacy. Eligible patients were identified via *ICD-10* codes—an imperfect although practical and frequently used approach that has strong negative predictive value.^25^ With limited patient-level data, we were unable to assess how these characteristics may have influenced our outcome, although a prior investigation found minimal added explanatory effect of clinical in addition to SDoH data, in terms of association with acute heart failure-related hospital admission.^26^ We also lacked data on in-hospital death, a competing risk for ED revisits and re-admissions.

Individual-level vulnerability may be more informative than area-level data, but the former are often inaccurately or incompletely collected.^27^,^28^ While ZIP codes may not align as well with population characteristics as other delineations (eg, census tracts), ZIP-level data are widely available and have been successfully implemented in outcome models related to acute heart failure.^29^ Cautious interpretation is needed, however, as area-level measures, including composite measures, may not align well with individual vulnerabilities.^30^

Finally, the suburban hospital (HVSH) had substantially different median SDI compared to the urban locations (13.5 versus 91–95). While the magnitude of SDI associations changed when HVSH patients were excluded, there was no loss of statistical significance. This could be due to sparse data in the mid-range of SDI scores, unmeasured hospital-level effects, or residual confounding. While not definitive, our results nonetheless provide an important first step toward elucidating the relationship between area-level social vulnerability and ED use related to acute heart failure.

## CONCLUSION

In this single-center, retrospective study from an urban health system, we found that increased community-level social vulnerability was associated with a higher number of 90-day revisits related to acute heart failure, but not with hospital admission. By identifying a risk factor for increased ED use, our results provide a foundation for understanding the complex decision of when and why to seek ED care for acute heart failure. This is a critical step toward developing targeted interventions to safely reduce ED revisits.

## Supplementary Information



## Figures and Tables

**Table 1:** Baseline Patient Characteristics.

Characteristic	N = 2,406^1^
Biological Sex	
Female	1,182 (49%)
Male	1,224 (51%)
Age	66.0 (57–75)
Hospital	
DRH	713 (30%)
HVSH	402 (17%)
HUH	603 (25%)
SGH	688 (29%)
Disposition	
Admit	1,683 (70%)
AMA	184 (7.6%)
Discharge	539 (22%)
Total Visits	1.0 (1–2)
SDI	94.0 (79–96)
Income Disparities	0.2 (0–0.3)
% Without Health Insurance	11.4 (9.3–13.2)
Median SBP	134.0 (133–135)
HTN Prevalence	46.5 (39.7–49.8)
Days Between Visits	53.5 (122–130)

1 n (%); Median (IQR); *DRH=*Detroit

Receiving Hospital; *SGH*= Sinai-Grace Hospital; *HUH*= Harper

University Hospital; *HVSH*= Huron Valley Sinai Hospital;

*AMA=*Against Medical Advice; *SDI*= Social Deprivation

Index; *SBP*=systolic blood pressure; *HTN*= hypertension

**Table 2:** Disposition at index vs. non-index visits

	Disposition at prior visit^1^			

	Index visit^2^	Admit N = 520^2^	AMA N = 71^2^	Discharge N = 156^2^
Disposition at current visit				
Admit	2,042 (72%)	434 (83%)	34 (48%)	67 (43%)
AMA	204 (7.2%)	29 (5.6%)	20 (28%)	11 (7.1%)
Discharge	576 (20%)	57 (11%)	17 (24%)	78 (50%)

1 The most recent visit compared to current visit

2 n (%)

Rows represent disposition at the most current visit; columns represent disposition at the immediate *prior visit*, except “index”, which, by definition, has no prior visit (e.g., of the 71 patients who left “AMA” at prior visits, 34 were admitted at the current visit, 17 were discharged, etc).

**Table 3:** Zero-Hurdle Poisson Model.

Characteristic	OR/RR^1^	95% CI	p-value
Zero-hurdle (Logistic) model			
SDI	1.53	1.08, 2.16	0.016
Hospital			
DRH	—	—	
HVSH	2.09	0.97, 4.53	0.061
HUH	1.34	0.99, 1.81	0.061
SGH	1.81	1.34, 2.44	<0.001
% without health insurance	0.94	0.79, 1.13	0.5
Age	0.89	0.80, 1.00	0.051
Median SBP (ZIP code level)	1.15	0.92, 1.43	0.2
Biological sex			
Female	—	—	
Male	1.28	1.03, 1.59	0.028
Count model (Positive Poisson)			
SDI	140	7.34, 2,672	0.001
Hospital			
DRH	—	—	
HVSH	22.9	3.52, 148	0.001
HUH	9.61	1.60, 57.6	0.013
SGH	11.1	1.86, 66.7	0.008
% without health insurance	0.64	0.49, 0.84	0.001
Age	0.68	0.60, 0.76	<0.001
Median SBP (ZIP code level)	1.47	1.17, 1.84	<0.001
Biological Sex			
F	—	—	
M	1.57	1.26, 1.96	<0.001
SDI ^*^ Hospital			
SDI ^*^ HVSH	0.02	0.00, 0.35	0.008
SDI ^*^ HUH	0.01	0.00, 0.25	0.004
SDI ^*^ SGH	0.02	0.00, 0.48	0.015

Continuous Variables are mean-centered and scaled

1 *OR*, odds-Ratio for Logistic Model; *RR*, relative risk for Count model; *CI*, confidence interval.

**Table 4:** Odds ratio of admission at any given visit.

Characteristic	Odds ratio	95% CI	p-value
Age	1.25	1.14, 1.37	<0.001
Days between visits	1.45	1.29, 1.62	<0.001
SDI	1.15	0.90, 1.48	0.3
% without health insurance	1.05	0.92, 1.20	0.4
Median SBP (Zip code level)	1.03	0.87, 1.21	0.8
Biological Sex			
Female	—	—	
Male	0.81	0.68, 0.96	0.016
Hospital			
DRH	—	—	
HUH	1.37	1.09, 1.71	0.007
HVSH	3.22	1.74, 5.95	<0.001
SGH	1.40	1.11, 1.76	0.004
Disposition at prior visit			
Index	—	—	
Admit	1.89	1.44, 2.47	<0.001
AMA	0.66	0.37, 1.15	0.14
Discharge	0.37	0.25, 0.55	<0.001
SD of Random Intercept	0.59		

Continuous Variables are mean-centered and scaled

*CI*, confidence interval; *DRH*, Detroit Receiving Hospital; *SGH*, Sinai-Grace Hospital; *HUH*, Harper University Hospital; *HVSH*, Huron Valley Sinai Hospital; *AMA*, Against Medical Advice; *SDI*, Social Deprivation Index; *SBP*, systolic blood pressure; *SD, Standard deviation*.
