# Supplementary material for: Social Deprivation Index Is Associated with 90-Day Emergency Department Revisits, but Not Admission, for Acute Heart Failure
Source: West J Emerg Med. 2026 Jul 16;27(4):1082–9. doi: 10.5811/westjem.61392 (PMC13436671; doi:10.5811/westjem.61392)
Supplement: Supplementary file 1 [file wjem-27-1082-s001.docx]

**SUPPLEMENT**

Table S1: Excluded diagnoses not consistent with Acute Heart Failure as primary reason for visit

| Diagnosis | n |
| --- | --- |
| 2019-nCoV acute respiratory disease | 12 |
| Acute bronchospasm | 1 |
| Acute embolism and thombos unsp deep veins of low extrem | 1 |
| Acute kidney failure, unspecified | 24 |
| Acute pancreatitis without necrosis or infection, unspecified | 1 |
| Acute pericarditis, unspecified | 1 |
| Adult failure to thrive | 1 |
| Altered mental status, unspecified | 3 |
| Anemia, unspecified | 4 |
| Bradycardia, unspecified | 1 |
| Cardiac arrest, cause unspecified | 3 |
| Cellulitis of right lower limb | 1 |
| Cerebral infarction, unspecified | 2 |
| Chronic kidney disease, unspecified | 1 |
| Chronic obstructive pulmonary disease w (acute) exacerbation | 34 |
| Disorder of kidney and ureter, unspecified | 1 |
| Dizziness and giddiness | 1 |
| Encephalopathy, unspecified | 2 |
| Encounter for issue of repeat prescription | 2 |
| Epigastric pain | 1 |
| Fracture of unsp part of neck of left femur, init | 1 |
| Gastrointestinal hemorrhage, unspecified | 1 |
| Hb-SS disease with crisis, unspecified | 3 |
| Hypo-osmolality and hyponatremia | 1 |
| Mech compl of other cardiac electronic device, init encntr | 3 |
| Nausea with vomiting, unspecified | 1 |
| Other pulmonary embolism without acute cor pulmonale | 1 |
| Other specified abnormalities of plasma proteins | 2 |
| Other specified soft tissue disorders | 1 |
| Pleurodynia | 1 |
| Pneumonia, unspecified organism | 19 |
| Poisoning by oth opioids, accidental (unintentional), init | 1 |
| ST elevation (STEMI) myocardial infarction of unsp site | 2 |
| Sepsis, unspecified organism | 3 |
| Syncope and collapse | 5 |
| Transient cerebral ischemic attack, unspecified | 1 |
| Unspecified abdominal pain | 1 |
| Unspecified asthma with (acute) exacerbation | 6 |
| Unspecified injury of head, initial encounter | 4 |
| Unspecified ovarian cyst, left side | 1 |
| Urinary tract infection, site not specified | 1 |

Table S2; Predicted counts of 90-day ED re-visits at varied SDI scores

| SDI* | Predicted | 95% CI |
| --- | --- | --- |
| Hospital: HVSH | | |
| 2 | 0.00 | [0.00, 0.00] |
| 79 | 0.07 | [0.01, 0.39] |
| 94 | 0.79 | [0.55, 1.13] |
| 96 | 1.09 | [0.86, 1.39] |
| 100 | 2.11 | [1.43, 3.10] |
| Hospital: DRH | | |
| 2 | 0.14 | [0.06, 0.31] |
| 79 | 1.52 | [0.81, 2.86] |
| 94 | 2.44 | [1.13, 5.26] |
| 96 | 2.60 | [1.18, 5.71] |
| 100 | 2.95 | [1.28, 6.76] |
| Hospital: HUH | | |
| 2 | 0.15 | [0.02, 1.06] |
| 79 | 0.64 | [0.45, 0.91] |
| 94 | 0.85 | [0.66, 1.09] |
| 96 | 0.88 | [0.67, 1.15] |
| 100 | 0.95 | [0.69, 1.30] |
| Hospital: SGH | | |
| 2 | 0.03 | [0.00, 0.30] |
| 79 | 0.74 | [0.52, 1.05] |
| 94 | 1.35 | [1.07, 1.71] |
| 96 | 1.47 | [1.13, 1.90] |
| 100 | 1.72 | [1.25, 2.38] |
| *Adjusted for: % without health insurance (11.30), age (65.6 years), median ZIP-code level SBP (134.48), averaged over levels of gender*  **SDI values are minimum, Q1, median, Q3, maximum* | | |

Associations between hospital and disposition at prior visit on the odds of admission.

Level-specific contrasts (marginal means) from ther mixed-effects model shown in **Figure S1** and **Figure S2**. Compared to Index visits, admission at the subsequent visit was more likely for patients who were admitted at their prior visit (OR 1.89 [CI 1.44-1.47] and less likely for patients who were discharged (OR 0.37 [CI 0.25-0.55]). Compared to admission at the prior visit, patients who left AMA were less likely to be admitted at the subsequent visit (OR 0.35, [CI 0.19-0.64]). For Index visits alone, the estimated marginal effect on admission for a +1, versus -1, SD change in SDI was OR 1.32 (CI 0.81-2.18).

Sensitivity analyses

With HVSH excluded, in the zero-hurdle model, the OR for SDI changed to 1.37 from 1.57; in the count component, RR for SDI*hospital interaction changed to 0.18 from 0.01 and 0.23 from 0.02 for HUH and SGH, respectively; in the mixed model, the OR for SDI changed to 1.03 from 1.15. Full model results are provided in the **Tables (S3 and S4)** below.

**Table S3: Zero-Hurdle Poisson Model Without HVSH Patients**

| **Characteristic** | **OR/RR***^1^* | **95% CI***^2^* | **p-value** |
| --- | --- | --- | --- |
| Zero-Hurdle (Logistic) Model | | | |
| **SDI** | 1.37 | 1.13, 1.67 | 0.002 |
| **% without Health Insurance** | 0.90 | 0.76, 1.06 | 0.2 |
| **Age** | 0.90 | 0.80, 1.01 | 0.080 |
| **Median SBP (ZIP code level)** | 1.12 | 0.97, 1.30 | 0.13 |
| **Biological Sex** |  |  |  |
| *F* | — | — |  |
| *M* | 1.31 | 1.03, 1.65 | 0.028 |
| **Hospital** |  |  |  |
| *DRH* | — | — |  |
| *HUH* | 1.37 | 1.01, 1.85 | 0.043 |
| *SGH* | 1.91 | 1.40, 2.59 | <0.001 |
| Count Model | | | |
| **SDI** | 1.17 | 0.94, 1.46 | 0.2 |
| **% without Health Insurance** | 0.82 | 0.65, 1.02 | 0.079 |
| **Age** | 0.84 | 0.75, 0.95 | 0.004 |
| **Median SBP (ZIP code level)** | 1.03 | 0.91, 1.17 | 0.6 |
| **Biological Sex** |  |  |  |
| *F* | — | — |  |
| *M* | 1.32 | 1.04, 1.66 | 0.020 |
| **Hospital** |  |  |  |
| *DRH* | — | — |  |
| *HUH* | 0.85 | 0.62, 1.15 | 0.3 |
| *SGH* | 1.04 | 0.79, 1.36 | 0.8 |
| Continuous Variables are mean-centered and scaled | | | |
| *^1^*Odds-Ratio (OR) for Logistic Model; Relative Risk (RR) for Count Model | | | |
| *^2^*CI = Confidence Interval  *F=*female; *M=*male; *DRH=*Detroit  Receiving Hospital; *SGH*= Sinai-Grace Hospital; *HUH*= Harper  University Hospital;*AMA=*Against Medical Advice; *SDI*= Social Deprivation  Index; *SBP*=systolic blood pressure | | | |

**Table S4: Odds Ratio of Admission at Any Given Visit Without HVSH Patients**

| **Characteristic** | **Odds Ratio** | **95% CI***^1^* | **p-value** |
| --- | --- | --- | --- |
| **Age** | 1.25 | 1.14, 1.37 | <0.001 |
| **Days Between Visits** | 1.45 | 1.29, 1.64 | <0.001 |
| **SDI** | 1.03 | 0.93, 1.16 | 0.5 |
| **% without Health Insurance** | 1.05 | 0.94, 1.17 | 0.4 |
| **Median SBP (Zip code level)** | 1.02 | 0.92, 1.13 | 0.7 |
| **Biological Sex** |  |  |  |
| *F* | — | — |  |
| *M* | 0.83 | 0.69, 1.00 | 0.046 |
| **Hospital** |  |  |  |
| *DRH* | — | — |  |
| *HUH* | 1.36 | 1.09, 1.71 | 0.007 |
| *SGH* | 1.39 | 1.11, 1.75 | 0.005 |
| **Disposition at Prior Visit** |  |  |  |
| *Index* | — | — |  |
| *Admit* | 2.03 | 1.52, 2.70 | <0.001 |
| *AMA* | 0.66 | 0.37, 1.15 | 0.14 |
| *Discharge* | 0.36 | 0.24, 0.55 | <0.001 |
| **SD of Random Intercept** | 0.58 |  |  |
| Continuous Variables are mean-centered and scaled | | | |
| *^1^*CI = Confidence Interval  *F=*female; *M=*male; *DRH=*Detroit  Receiving Hospital; *SGH*= Sinai-Grace Hospital; *HUH*= Harper  University Hospital; *AMA=*Against Medical Advice; *SDI*= Social Deprivation  Index; *SBP*=systolic blood pressure; *SD=Standard deviation* | | | |
